# Accumulated unhealthy behaviors and psychosocial problems in adolescence are associated with labor market exclusion in early adulthood – a northern Finland birth cohort 1986 study

**DOI:** 10.1186/s12889-020-08995-w

**Published:** 2020-06-05

**Authors:** Eveliina Heikkala, Leena Ala-Mursula, Simo Taimela, Markus Paananen, Eeva Vaaramo, Juha Auvinen, Jaro Karppinen

**Affiliations:** 1 Medical Research Center Oulu, University of Oulu and Oulu University Hospital, PO Box 5000, 90014 Oulu, Finland; 2 Rovaniemi Health Center, Koskikatu 25, 96200 Rovaniemi, Finland; 3 Center for Life Course Health Research, University of Oulu, Aapistie 5B, 90150 Oulu, Finland; 4 Department of Orthopedics and Traumatology, Helsinki University Hospital, Töölö hospital, Topeliuksenkatu 5, 00260 Helsinki, Finland; 5 Clinicum, Department of Orthopedics and Traumatology, University of Helsinki, PO Box 266, 00029 HUS Helsinki, Finland; 6 Intrastructure for Population Studies, Faculty of Medicine, University of Oulu, Aapistie 5B, 90150 Oulu, Finland; 7 Oulunkaari Health Center, Piisilta 1, 90110 Ii, Finland; 8 Finnish Institute of Occupational Health, Aapistie 1, 90220 Oulu, Finland

**Keywords:** Adolescent, Health behavior, Psychosocial problem, Labor market, Multisite musculoskeletal pain, Young adulthood, Latent class analysis, Follow-up, Cohort study

## Abstract

**Background:**

The relevance of health-related behaviors to exclusion from the labor market in early adulthood remains poorly studied in relation to the magnitude of the problem. We explored whether adolescents’ accumulated unhealthy behaviors and psychosocial problems are associated with later labor market exclusion, and whether multisite musculoskeletal pain (MMSP) impacts these relations.

**Methods:**

We gathered questionnaire data on unhealthy behaviors and psychosocial problems and MMSP among adolescents aged 15 to 16 belonging to the Northern Finland Birth Cohort 1986. The findings were combined with registry data on unemployment, employment and permanent work disability during a five-year follow-up between the ages of 25 and 29 (*n* = 6692). In the statistical modeling we used education, family leave and socioeconomic status of childhood family as potential confounders, as well as latent class and logistic regression analyses.

**Results:**

The Externalizing behavior cluster associated with over one year of unemployment (RR 1.64, CI 1.25–2.14) and permanent work disability (OR 2.49, CI 1.07–5.78) in the follow-up among the men. The Sedentary cluster also associated with over one year (RR 1.41, CI 1.13–1.75) and under one year of unemployment (RR 1.25, CI 1.02–1.52) and no employment days (RR 1.93, CI 1.26–2.95) among the men. Obese male participants were at risk of over one year of unemployment (RR 1.50, CI 1.08–2.09) and no employment days (RR 1.93, CI 1.07–3.50). Among the women, the Multiple risk behavior cluster related significantly to over one year of unemployment (RR 1.77, CI 1.37–2.28). MMSP had no influence on the associations.

**Conclusions:**

Unhealthy behavior patterns and psychosocial problems in adolescence have long-term consequences for exclusion from the labor market in early adulthood, especially among men. Simultaneously supporting psychological well-being and healthy behaviors in adolescence may reduce labor market inclusion difficulties in the early phase of working life.

## Background

It is not easy for young people to enter the labor market today [1]. For instance, in Europe, 18 % of today’s 15–24-year-olds [1] and over 11 % of 25–29-year-olds [2] have no job, and around one sixth can be regarded as outsiders of both the labor and education market [1, 2]. Even though temporary unemployment or an unstable employment situation may be part of the ordinary pathway to labor market inclusion, nearly one-third of unemployed young people report long-term unemployment [2].

Labor market inclusion problems seem to expose the young to poorer general health and quality of life, risk behaviors and sickness absences [3]. Young people who do not find a job during the early years [4], who undergo long or repeated unemployment periods [5, 6] or who have low-level education [2, 7] are likely to be at a disadvantage in terms of health consequences. Unemployment is not only a financial problem for the young themselves but also for societies [8]. Aging western societies clearly need to extend working careers to support the sustainability of national economies; importantly, not only in the later stages but also at the beginning of the working career. The difficulties young people face when entering the labor market are of population-level relevance.

The early precursors of labor market inclusion remain less extensively studied, but research on the role of health problems in poor inclusion is gradually increasing. The most serious health problems, such as chronic pediatric illnesses and disabilities occurring in childhood and adolescence, have indeed been recognized as risks of later exclusion from the labor market [9]. In addition, some predictors of poorer health, such as unhealthy behaviors or psychosocial problems, appear to be associated with unemployment [10–12] and to play a significant role in later poorer participation in the labor market [13–18]. These health-related determinants have mainly been studied one at a time. Some recent studies have pointed out that diverse risk factors accumulate [13, 19, 20], suggesting that young people’s risk behaviors, mental health, overweight/obesity, and physical inactivity are jointly associated with labor market outcomes [13, 18, 21]. However, studies aiming for a deeper understanding of health-related exclusion from working life by means of detecting different profiles of accumulating health risks among young people are so far lacking.

Pain in the musculoskeletal system is known to associate with labor market exclusion in adulthood. It is listed as one of the major causes of disability pension [22] and is believed to reduce work ability [23]. Of all musculoskeletal pains, multisite musculoskeletal pain (MMSP) seems to be the most detrimental, as an increasing number of pain sites leads to a higher risk of disabilities in the general working population [23, 24]. Whether this applies to young people is not known. Moreover, Moreover, adolescent MMSP was associated with clusters of unhealthy behaviors and psychosocial problems in our previous study [25].

To date, no study has longitudinally explored the associations of the accumulation of unhealthy behaviors and psychosocial problems in the pre-employment stage of life with labor market exclusion later in early adulthood, nor the role of multisite pain in these potential relations. Thus, we set up a study in a large birth cohort to investigate whether 1) discrete patterns of smoking, physical activity level, sleeping time, sedentary behavior, overweight/obesity, and externalizing and internalizing problems surveyed at the age of 16 would be associated with later nationally registered unemployment, employment and permanent work disability figures during a five-year follow-up period between the ages of 25 and 29, and 2) whether MMSP would modify these possible associations. In the analyses, we used earlier formed health behavior and psychosocial clusters [19], and considered childhood family socioeconomic status (SES), educational level and family leaves, retrieved from the registers, as potential confounding factors.

## Methods

### Study population

The Northern Finland Birth Cohort (NFBC1986) is a large mother-child birth cohort collected from the two northernmost provinces of Finland, which originally consisted of 9432 live-born children. The baseline data of the present study were gathered via a questionnaire mailed to the 15- to 16-year-old eligible adolescents belonging to the NFBC1986 between 2001 and 2002 (*n* = 9215). A total of 7344 adolescents replied, of whom 6749 had responded to questions on health behaviors and psychosocial factors and MMSP. At the same time, another survey was delivered to the participants’ parents to collect information on childhood family SES.

When the members were aged 25–29, the questionnaire data were combined with five-year (2011–2015) coverage of nationally registered data from the Finnish Centre for Pensions, Statistics Finland and The Social Insurance Institution of Finland, via national personal identification numbers. The data included individually calculated days in employment and unemployment, family leaves and permanent work disability, as well as data on the highest education level.

Our final study population consisted of 3180 men and 3512 women who were alive and had provided health behavior, psychosocial, and MMSP information at 16 years, and had available register data.

### Outcome variables

#### Unemployment

We calculated the total number of days for which each participant had received unemployment benefit between 2011 and 2015. Unemployment days were then categorized into three groups: 1) no unemployment days, 2) under one year of unemployment (in Finland, a person receives unemployment allowance for five days a week: under one year = less than 260 days), and 3) over one year of unemployment. The classification of the groups was based on the general policy of over one year of unemployment being a long-term situation [1, 2]. We excluded the participants (*n* = 72) who had received either disability pension or rehabilitation subsidy (= fixed-term disability pension) as proxy measures of permanent work disability from the unemployment analyses because they had not been in the labor market during the entire five-year follow-up.

#### Employment

We calculated the total number of days for which a participant had received income and applied the same exclusion criteria as that for unemployment. Three categories were formed: a) no employment days between the ages of 25 and 29, b) less than four years of employment, and c) over four years of employment during the five-year follow-up.

#### Permanent work disability

As permanent work disability is rare before the age of 30, we detected this early exclusion from the labor market due to work disability on the basis of receipt of any type of full- or part-time disability pension or rehabilitation subsidy (=fixed-term disability pension) during the follow-up, and dichotomized this outcome as yes vs. no. With very few exceptions, these benefits are granted only after a sickness absence has lasted for at least one year.

In addition to unemployment, employment and permanent work disability, we considered assessing sickness absences. However, the register data of The Social Insurance Institution of Finland only cover sickness allowances exceeding the waiting periods paid by the employers. These sickness absence periods, most often paid for ten working days, do however vary depending on work contracts, and cannot be separated from paid employment days. Since accurate assessment was beyond our reach, we did not include sickness absence days in the present study.

### Explanatory factors at 16 years

#### Factors used in latent class analysis

##### Physical activity

Weekly duration of moderate-to-vigorous activity causing shortness of breath and sweating, performed outside school hours, was categorized as follows: ≥3 h, active; 2–3 h, moderately active; and ≤ 1 h, inactive.

##### Smoking

Smoking was categorized as (1) non-regular smoker, (2) 0.1–1.0 pack-year and (3) over 1.0 pack-years, by the age of 16 [26]. One pack-year equaled 15 cigarettes smoked per day per year.

##### Sleeping time

For hours spent sleeping per day, three patterns were formed: 1) < 8 h per day, 2) 8–9 h per day and 3) > 9 h per day.

##### Sitting hours

A sum of total hours sitting while watching television, reading books or magazines, working on a computer or playing video games, or doing other sedentary activities was trichotomized into: < 4.1 h, 4.1–7.9 h, and > 7.9 h per day among men. For women we used a continuous variable.

##### Overweight/obesity

Weight and height were measured in a health examination at 16 years and were calculated as body mass index (BMI). For those not participated in the health examination (12% of adolescents), self-reported values were used. BMI was addressed as a continuous variable. International Obesity Task Force age-specific cut-off points for BMI provided the scales for overweight: 23.90–28.88 kg/m^2^ for men and 24–29.43 kg/m^2^ for women; and for obesity among men: 28.88 kg/m^2^ and among women: 29.43 kg/m^2^ [27].

##### Externalizing and internalizing problems

We assessed the psychosocial symptoms during the preceding six months using the Youth-Self Report (YSR) questionnaire. The questionnaire consisted of 105 items, for which the individuals rated themselves on a scale of 0–2 (0 = not true, 1 = somewhat or sometimes true, and 2 = very true or often true). The scores for the items were summed up to obtain summary scores for eight subscales, of which anxious/depressed symptoms, withdrawn/depressed symptoms and somatic complaints constituted the internalizing scale, whereas rule-breaking and aggressive behaviors formed the externalizing scale. The calculated scores were trichotomized into normal range, borderline range, and clinical range [28]. The last two established the “problem range” for the analyses. Those with over eight missing responses to the YSR were excluded. In other cases, the mean value of the specific scale was applied for substituting the missing values.

In the selection procedure of variable classification, all the variables we used were first applied in Latent class analysis (LCA) as continuous, but this led to a massive loss of information and group sizes that were too small. After this we tried several different variations of categorized and continuous variables. The solution utilized in the present study lost the least information and was easy to interpret [19].

#### Multisite musculoskeletal pain

Four subgroups of different pain sites during the preceding six months at 16 years were constituted in relation to pain area (low back, shoulder, neck/occipital and peripheral area). Having pain in two or more sites was considered “MMSP” and no pain or pain in one site as “no multisite pain”.

### Confounding factors

Childhood family SES at the age of 16 was indicated by parents’ occupational status and was categorized as follows: (a) higher clerical employees; (b) self-employed; (c) lower clerical employees; (d) workers, and (e) students, pensioners, unemployed or unknown. The fathers’ responses were prioritized.

Data on participants’ lifetime highest education level by the age of 29 was obtained from national registers and was categorized according to the following degrees: 1) compulsory, 2) secondary (upper secondary or vocational school) and 3) tertiary (university or university of applied sciences).

To indicate participants’ own family status during the follow-up, we applied the registered days from The Social Insurance Institution of Finland for which a person had received any maternity, paternity or parental allowance or home care support between the ages of 25 and 29. There are no deductible times for these allowances, but depending on work contracts, some periods of maternity leave can be paid by employers. Therefore, any family leave-based registered data were dichotomized as yes vs. no.

### Statistical analysis

We used LCA to identify a set of discrete latent groups of adolescents based on their health behavior and psychosocial characteristics. This statistical method aims to seek uncovered but homologous subgroups of participants from the original heterogeneous population by identifying similar patterns of response items to studied variables and classifying individuals into a most probable group (=cluster) on the basis of the posterior probabilities of membership.

We assessed cluster models of one to seven clusters and determined the optimal number of classes using a number of fit indices: Bayesian information criterion (BIC), the sample-size adjusted BIC (SSBIC) and the Akaike information criterion (AIC), entropy, and Lo-Mendell-Rubin test (LRT) (Table 1). In LCA, low values of BIC, AIC, and SSABIC, a high entropy, and a statistically significant LRT value reflect the best model fit. Furthermore, the conceptual meaningfulness and cluster sizes are also relevant. Among the men, the lowest BIC value, and the interpretability and size of the clusters favored a four-cluster model. Among the women, a four-cluster solution had the lowest BIC value, low AIC and SSABIC values, high entropy, and statistically significant LRT value, which stressed the superiority of one model over other model solutions [19].

**Table 1:** Fit statistics for a one-cluster model through to a seven-cluster-model among men and women

MEN	AIC	BIC	SSABIC	Entropy	LRT
1-cluster model	38,310.975	38,384.202	38,346.073	N/A	N/A
2-cluster model	37,755.683	37,902.138	37,825.879	0.811	0.1190
3-cluster model	37,390.284	37,609.967	37,495.578	0.784	0.3129
4-cluster model	37,275.750	37,568.660	37,416.142	0.644	0.0008
5-cluster model	37,236.918	37,603.055	37,412.408	0.833	< 0.001
6-cluster model	37,209.182	37,648.546	37,419.770	0.679	0.2307
7-cluster model	37,140.859	37,653.450	37,386.545	0.822	1.0000
WOMEN					
1-cluster model	26,843.382	26,908.612	26,870.482	N/A	N/A
2-cluster model	25,793.163	25,935.439	25,862.357	0.654	< 0.0001
3-cluster model	25,706.917	25,917.237	25,809.203	0.634	0.0141
4-cluster model	25,328.046	25,606.412	25,463.425	0.804	0.0050
5-cluster model	25,295.221	25,641.632	25,463.692	0.707	0.4722
6-cluster model	25,255.253	25,669.709	25,456.817	0.762	0.6387
7-cluster model	25,283.651	25,766.152	25,518.307	0.824	0.1721

AIC = Akaike Information Criteria

BIC=Bayesian Information Criteria

SSABIC=Sample Size Adjusted Bayesian Information Criteria

LRT = *p*-value for the Lo-Mendell-Rubin Likelihood Ratio Test

We conducted cross-tabulations including Chi square tests and logistic regression analyses to analyze potential associations between LCA clusters and labor market outcomes. To evaluate the role MMSP in these possible associations, we utilized logistic regression analyses and modeled an interaction term of LCA clusters and MMSP. We used multinomial logistic regression analyses, with risk ratios (RR) for unemployment and employment, and binomial logistic regression analyses, with odds ratios (OR) for permanent work disability. We adjusted the associations for childhood family SES, educational level and family leaves. To evaluate attrition, we compared the levels of childhood family SES at 16 years among those who provided data on baseline factors to the corresponding figures of participants not included in the cluster analyses. The significance level of the *p*-value was set at 0.05, and 95% confidence intervals were calculated for RR and OR derived. We used SPSS Version 25.0 and STATA Version 13.1 in the analyses.

## Results

### Descriptive information

Among the men, 21% and among the women, 22% had been unemployed for over a year, whereas 24% of the men and 28% of the women had been unemployed for less than one year during the follow-up period (*p* < 0.001; Table 2). More men than women recorded employment days of over four years (62% vs. 50%, *p* < 0.000), but we found no difference in having no employment days (5 and 6%). Seventy-two of the participants had received disability pension or cash rehabilitation benefits between the ages of 25 and 29. A nearly two-fold number of women compared to the men had family leave days (48% vs. 27%, *p* < 0.000), and over 90% of both genders were educated. MMSP was reported by 43% of the men and 64% of the women at 16 years (*p* < 0.000).

**Table 2:** Demographic characteristics of men and women related to unemployment, employment, permanent work disability, family leaves and education during five-year follow-up at 25 to 29 years, and latent class clusters and multisite musculoskeletal pain at 16 years

	MEN N (%)	WOMEN N (%)	*P* value (x^2^)
Unemployment*			< 0.001
No unemployment	1810 (55)	1830 (50)	
Under one year	799 (24)	999 (28)	
1 to 2 years	299 (9)	400 (11)	
2 to 3 years	186 (6)	241 (7)	
3 to 4 years	131 (4)	111 (3)	
4 to 5 years	81 (2)	45 (1)	
Employment*			< 0.001
No employment	151 (5)	201 (6)	
Under one year	204 (6)	298 (8)	
1 to 2 years	208 (6)	352 (10)	
2 to 3 years	248 (8)	441 (12)	
3 to 4 years	423 (13)	523 (14)	
4 to 5 years	2072 (62)	1811 (50)	
Family leaves*			< 0.001
Yes	902 (27)	1757 (48)	
No	2404 (73)	1869 (52)	
Permanent work disability			0.532
Yes	37 (1)	35 (1)	
No	3306 (99)	3626 (99)	
Highest education			< 0.001
Compulsory	260 (8)	146 (4)	
Secondary	1862 (56)	1607 (44)	
Tertiary	1221 (37)	1908 (52)	
Socioeconomic status of childhood family**			0.730
Higher clerical employees	242 (7)	240 (7)	
Self-employed	575 (17)	630 (17)	
Lower clerical employees	501 (15)	539 (15)	
Workers	1333 (40)	1459 (40)	
Students, pensioners, unemployed or unknown	692 (21)	793 (22)	
MMSP at 16**			< 0.001
No pain or one pain-site	1862 (57)	1300 (36)	
Two or more pain sites	1392 (43)	2296 (64)	
Clusters**			< 0.001
Cluster 1	472 (14)	540 (15)	
Cluster 2	886 (27)	425 (12)	
Cluster 3	258 (8)	239 (7)	
Cluster 4	1686 (51)	2386 (66)	

*those with disability days are not included

**questionnaire-based information

MMSP = multisite musculoskeletal pain

The ‘Workers’ group was the most prevalent socioeconomic status group of childhood family among both men and women (40%), followed by ‘students, pensioners, unemployed or unknown’ (21% for men, 22% for women), ‘self-employed’ (17%), ‘lower clerical employees’ (15%) and ‘higher clerical employees’ (7%). In the comparison analyses of the cluster participants and others belonging to the NFBC1986, slightly more of the other NFBC1986 members than the cluster participants belonged to ‘students, pensioners, unemployed or unknown’ category (data not shown).

### Latent cluster demographics

**Fig. 1:**
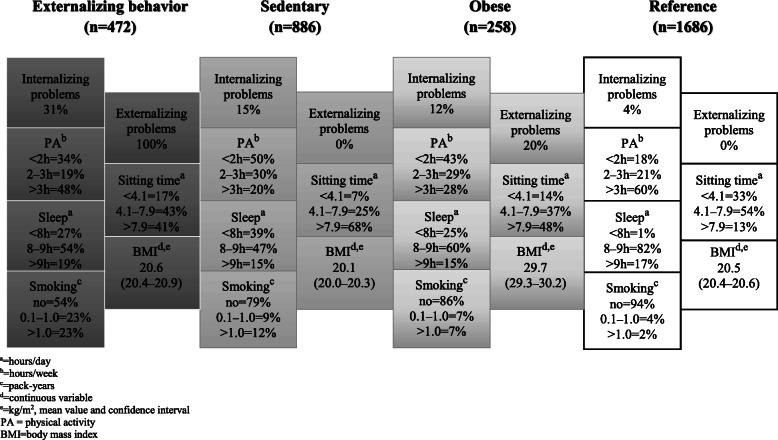
Frequency rates and mean values of unhealthy behaviors and psychosocial problems within clusters among men (*n* = 3302)

Among the men, a high likelihood of both internalizing and externalizing problems led Cluster 1 to be named the *Externalizing behavior* cluster (Fig. 1, prevalence rate 14%). These adolescents were also most likely to smoke. Cluster 2 (*Sedentary,* 27%) represented men with the highest probability of physical inactivity and sedentary behavior as well as short sleeping time. Cluster 3 was labelled *Obese* (8%) because the participants’ average BMI was 29.7. Sedentary behavior and physical inactivity were common within this cluster. Cluster 4 was the largest cluster, with a prevalence rate of 51%. As in the case of the women, this was named “*Reference*”, based on the overall favorable distribution of the included factors.

**Fig. 2:**
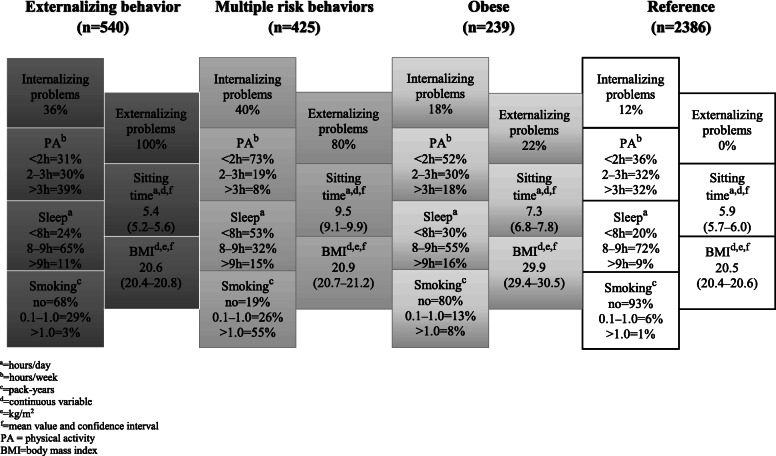
Frequency rates and mean values of unhealthy behaviors and psychosocial problems within clusters among women (*n* = 3590)

Among the women, Cluster 1 (*Externalizing behavior*, Fig. 2, prevalence rate 15%) comprised adolescents with externalizing problems and quite a high likelihood of internalizing problems. They were physically the most active. The profile of the respondents in Cluster 2 (*Multiple risk behaviors*, 12%) showed a pattern of high levels of all the unhealthy behaviors. In addition, psychosocial problems appeared to emerge among these young people. Cluster 3 (*Obese*, 7%) included women with a high BMI and a relatively high probability of physical inactivity and average sitting time. Cluster 4 (*Reference*, 66%) showed the overall favorable distribution of the factors studied.

### LCA clusters and unemployment

Among the men, the participants in all clusters except the Reference cluster were more likely to have been unemployed for over a year (Externalizing behavior: RR 1.64, CI 1.25–2.14; Sedentary: RR 1.41, CI 1.13–1.75; Obese: RR 1.50, CI 1.08–2.09; Table 3). Among the women, belonging to the *Multiple risk behaviors* (RR 1.77, CI 1.37–2.28) cluster associated significantly with over one year of unemployment. The men in the *Sedentary* cluster were at risk of under one year of unemployment (RR 1.25, CI 1.02–1.52).

**Table 3:** Logistic regression analysis for associations of latent class clusters at 15–16 years with cumulative unemployment and employment days, and permanent work disability at 25–29 years among both genders

	Unemployment (*n* = 6769) RR (CI)			Employment (*n* = 6769) RR (CI)			Permanent work disability (*n* = 6835) OR (CI)	
*MEN*	Over one year of unemployment	Under one year of unemployment	No unemployment days	No employment days	Under four years of employment	Over four years of employment	Yes	No
Externalizing behavior	**1.64** (1.25–2.14) (*n* = 123)	1.20 (0.92–1.56) (*n* = 106)	1.0	1.26 (0.73–2.15) (*n* = 25)	1.25 (0.99–1.58) (*n* = 160)	1.0	**2.49** (1.07–5.78) (*n* = 11)	1.0
Sedentary	**1.41** (1.13–1.75) (*n* = 202)	**1.25** (1.02–1.52) (*n* = 225)	1.0	**1.93** (1.26–2.95) (*n* = 54)	1.08 (0.90–1.30) (*n* = 286)	1.0	0.97 (0.38–2.49) (*n* = 7)	1.0
Obese	**1.50** (1.08–2.09) (*n* = 71)	0.86 (0.61–1.22) (*n* = 48)	1.0	**1.93** (1.07–3.50) (*n* = 19)	1.07 (0.80–1.44) (*n* = 84)	1.0	1.74 (0.55–5.47) (*n* = 4)	1.0
Reference	1.0	1.0		1.0	1.0		1.0	
*WOMEN*								
Externalizing behavior	1.18 (0.93–1.51) (*n* = 122)	0.90 (0.72–1.13) (*n* = 137)	1.0	0.78 (0.49–1.26) (*n* = 25)	0.91 (0.75–1.11) (*n* = 229)	1.0	2.06 (0.86–4.91) (*n* = 8)	1.0
Multiple risk behaviors	**1.77** (1.37–2.28) (*n* = 148)	0.91 (0.69–1.21) (*n* = 85)	1.0	0.86 (0.55–1.35) (*n* = 34)	1.04 (0.83–1.30) (*n* = 194)	1.0	1.81 (0.73–4.47) (*n* = 8)	1.0
Obese	1.37 (0.99–1.90) (*n* = 69)	0.81 (0.57–1.14) (*n* = 52)	1.0	1.07 (0.61–1.87) (*n* = 19)	0.96 (0.72–1.27) (*n* = 102)	1.0	0.45 (0.06–3.48) (*n* = 1)	1.0
Reference	1.0	1.0		1.0	1.0		1.0	

Adjusted for education level, family leaves and childhood family SES

Bolded values are statistically significant

RR = risk ratio

OR = odds ratio

CI = confidence interval

### LCA clusters and employment

For the men, belonging to the *Sedentary* (RR 1.93, CI 1.26–2.95) and *Obese* (RR 1.93, CI 1.07–3.50) clusters significantly increased the risk of having no employment days (Table 3). We observed no significant associations between the clusters and employment days among the women.

### LCA clusters and permanent work disability

In the *Externalizing behavior* cluster, the men (OR 2.49, CI 1.07–5.78) were at risk of permanent work disability during follow-up (Table 3). Among the women, we found no significant associations between LCA clusters and permanent work disability.

### Multisite musculoskeletal pain

MMSP had no significant association with any outcome variable studied (Table 4). We also added MMSP to the logistic regression analysis models of clusters and outcomes, formed an interaction term of LCA clusters and MMSP and included the interaction term in the analyses. The *p*-values of the interaction terms were high, emphasizing that MMSPs do not play an independent role in the outcomes.

**Table 4:** Logistic regression analysis for associations of multisite musculoskeletal pain at 16 years with cumulative unemployment, and employment days, and permanent work disability at 25–29 years among both genders

	MEN		WOMEN	
	Multisite musculoskeletal pain (RR/OR, CI)	No pain/one pain-site	Multisite musculoskeletal pain (RR/OR, CI)	No pain/one pain-site
Over 1 year of unemployment*	0.86 (0.72–1.04) (*n* = 266)	1.0	1.02 (0.86–1.22) (*n* = 502)	1.0
Under 1 year of unemployment*	0.96 (0.81–1.14) (*n* = 333)	1.0	0.93 (0.79–1.10) (*n* = 614)	1.0
No unemployment*	1.0		1.0	
No employment days*	0.79 (0.54–1.15) (n = 50)	1.0	0.97 (0.71–1.33) (n = 122)	1.0
Under 4 year of employment*	1.04 (0.89–1.21) (*n* = 457)	1.0	1.06 (0.92–1.22) (*n* = 1021)	1.0
Over 4 year of employment*	1.0		1.0	
Permanent work disability**	1.34 (0.69–2.62) (*n* = 17)	1.0	1.46 (0.70–3.06) (n = 25)	1.0
No permanent work disability**	1.0		1.0	

Adjusted for education level, family leaves and childhood family SES

RR = risk ratio

OR = odds ratio

CI = confidence interval

*=*N* = 6782

**=*N* = 6852

## Discussion

We explored the associations of four distinct latent class clusters of 16-year-old adolescents’ health behaviors and psychosocial factors with later register-based employment, unemployment and permanent work disability figures during a five-year follow-up at the age of 25 to 29. We found that the groups of adolescents with unhealthy behaviors and psychosocial problems were at a significantly higher risk of future exclusion from the labor market. MMSP did not associate with the observed relations.

Nearly half of the participants had registered unemployment days between the age of 25 and 29, and almost 50% of these had received unemployment benefit for over a year. Unfortunately, such high rates are not specific to this cohort. This indicates that finding ways with which to ease young people’s entry into the labor market could hold huge potential for extending working careers in the early phase. Internationally, in previous unemployment studies from the 1980s to the early 2000s, the prevalence of young adults experiencing at least one day of unemployment has varied between 42 and 70%, depending on the country, the age of the population and the measured time point [12, 17, 29]. A large cohort study of all Finnish young adults born in 1987 reported that almost 60% of the study sample had received unemployment benefits by the age of 25 [30], which, like our results, indicates a high prevalence of unemployment between adolescence and early adulthood in Finland. This may partly be related to difficulties in the transition from education to working life. One may not find a job straight after graduation and be in short-term or temporary employment in the meantime. Starting in seasonal jobs only and otherwise receiving unemployment benefits is also quite common. According to a recent International Labour Organization report, it takes over 17 months on average to find a long-term job after school in Europe [1].

The relevance of accumulated unhealthy behaviors and/or psychosocial problems during a pre-employment period on labor market exclusion over 10 years later appears greater than that of having none, one or even two unhealthy behaviors. Our finding gives further support to the relevance of the accumulation of health-related risks in terms of societal functioning. A study by Rodwell et al. [18] found that adolescents with both mental and externalizing disorders and cannabis use had a probability of 20% of not being in education or employment compared to a probability of 5% among those with no risk factors. Analogously, a Finnish study [13] found that young adults regarded as persistent heavy drinkers, smokers and physically inactive between the ages of 27 and 33 had the lowest earning levels and highest amount of unemployment months in comparison to their counterparts with none, one or two risky behaviors. Among adults, a dose-response relationship between an increasing number of unhealthy behaviors and the number of sickness absence days was found [31].

The co-occurrence of physical inactivity, sedentary behavior, short sleeping time, and obesity was associated with poor labor market inclusion in the current study among the men. In previous longitudinal studies, adolescents’ sedentary behavior has correlated with unemployment in adulthood [17] and young adults’ physical inactivity with reduced income and increased unemployment in later adulthood [13]. Insufficient sleep tends to relate to school performance [32], which in turn is associated with employment [7]. Sedentary behaviors with inadequate sleeping time and lack of exercise might impact on general well-being and productivity, which may reflect negatively on working life. As regards obesity, a cross-sectional study of 23-year-olds found it to associate with earnings [33] and another study found obesity to associate with long-term unemployment among 18–34-year-old women [34]. Laitinen et al. [29], however, observed no relation between adolescent obesity and history of unemployment at 31 years in a longitudinal setting. Perhaps it obesity combined with inactive lifestyle among adolescent men has a detrimental impact, as in our *Obese* cluster, the prevalence of physical inactivity and sitting time was also high. In a large Swedish study, obese adolescents who were also unfit at 16 to 19 years had the highest risk of later receipt of disability pension after a median follow-up of 28 years [21].

The men in the *Externalizing behavior* cluster were at risk of exclusion from the labor market. In contrast, belonging to the *Externalizing behavior* cluster did not relate to such exclusion among the women. Yet the cluster profiles of the genders were quite similar. According to the literature, psychosocial problems during adolescence are among the most significant determinants of unemployment [15, 35], labor market participation/marginalization in a broad sense [9, 16], and low earnings [9, 36] and some suggestions of a combined impact of several psychosocial problems has also been noticed in lower academic achievement [20]. A few possible explanations for the gender difference emerged in our study. One could be that the women with *Externalizing behaviors* were physically more active than the other women. A high level of physical activity already during adolescence has been found to reduce job strain [37] and increase earnings [38] in adulthood, and to increase/maintain work ability in the general population [39]. On the other hand, the relevance of psychosocial problems to labor market inclusion might depend on concurrent unhealthy behaviors, as the *Multiple risk behavior* cluster associated significantly with over one year of unemployment, and the men in the *Externalizing behavior* class smoked the most. Although the women in the *Externalizing behavior* cluster also included smokers, the prevalence of smoking was twice as high in the *Multiple risk behaviors* cluster.

Contrary to previous findings among the adult population [23, 24], MMSP did not play an independent role in labor market inclusion among young people, regardless of the associations of the clusters with the labor market outcomes. This observation may be related to the variable we used, as MMSP was described as having two or more sites during the preceding six months. Perhaps a narrower time period, such as experiencing MMSP during the previous month, would have led to positive associations [23, 24]. Taking into account pain intensity or dysfunctions caused by pain might also have influenced the results, but these aspects of pain were unfortunately not elicited in the questionnaire at 16 years. Nonetheless, the findings of our study emphasize the magnitude of co-occurring unhealthy behaviors and psychosocial problems in relation to labor market exclusion among young people.

The major strengths of the present work include its large, representative birth cohort followed until early adulthood, the fact that it covered all branches of the economy and occupations in the general population and avoided biases originating from fluctuations in macroeconomic cycles, its reliable registry-based outcomes with no recall bias and its prospective study design providing multiple aspects of participation in the labor market. From the Finnish registers, we were able to exploit data not only on unemployment, employment and permanent pension benefits, but also on education and family leaves as potential confounders. The follow-up period, based on the age span of this birth cohort, is a relevant one, as it adequately reflects the fact that today, young people remain in education for longer and it takes more time to enter the labor market [1]. This is the reason why large statistics have also used a similar age range in their analyses on youth employment [1, 2].

However, our results should be interpreted in the light of some limitations. The main limitation is built into the structure of the registered data regarding days in paid employment, as some of the paid days are in fact sickness absence or maternity leave days, and the amounts vary depending on the work contracts. Moreover, they cannot be separated from true working days. The results regarding employment days are therefore slight overestimates. In addition, the data do not differentiate whether the paid days result from working just a few hours or full time. Nevertheless, the paid days represent the periods during which a true connection to working life has existed. Interpreting unemployment days is quite straightforward, although some of such days arise from temporary lay-offs despite valid work contracts and although some students may have received unemployment benefits during school breaks. Our dichotomized estimate of permanent work disability during the five-year follow-up is a conservative one, since it is not possible to accurately detect the preceding period of sick leave. Overall, register-based unemployment, and employment and disability days illustrate labor market integration to a major extent and are easy to interpret. As another limitation, we have no survey-based follow-up data in adulthood on health behavior and psychosocial problems within this cohort so far. Our results stem from an ethnically homogeneous birth cohort in a Northern European welfare country, and in terms of generalizability of results, context-relatedness in studies is always an issue that relates health to labor market outcomes. Although the birth cohort setting enables the detection of longitudinal consequences of pre-employment behavior and labor market inclusion, the sizes of such associations may vary inter-culturally and in subpopulations, and warrant further studies in other cohorts.

## Conclusions

To the best of our knowledge, this is the first study using risk clusters when linking accumulated unhealthy behaviors and psychosocial problems in adolescence with registered poorer social functioning a decade later, when the participants are in their late twenties. The window of opportunity for preventing the young being excluded from the labor market because of health issues seems to emerge already in adolescence. As young people who are excluded from the labor market comprise a difficult target for effective health prevention interventions [40] and the evidence on the effectiveness of later health prevention both at workplaces [41] and among the unemployed [42] remains at best moderate, more feasible interventions are urgently needed. Supporting adolescents’ healthy lifestyles and psychological well-being could nevertheless foster young people’s labor market inclusion, thus extending working careers at the beginning of working life. Maintaining and developing healthy behaviors has shown to associate with less challenges in maintaining work ability among adults [43]. Therefore, future research should verify our finding of the role of accumulated adolescents’ behaviors in labor market outcomes by following the later trajectories of health behaviors and psychosocial factors during adulthood in relation to sustained inclusion in the labor market.

## Data Availability

Data are available from the Northern Finland Birth Cohort Data and Publication Committee for researchers who meet the criteria for access to confidential cohort data. Please, contact the NFBC project center (NFBCprojectcenter@oulu.fi) and visit the cohort website (www.oulu.fi/nfbc) for more information.

## Abbreviations

MMSP: Multisite musculoskeletal pain
SSBIC: Sample-size adjusted BIC
NFBC1986: Northern Finland Birth Cohort 1986
AIC: Akaike information criterion
BMI: Body mass index
LRT: Lo-Mendell-Rubin test
YSR: Youth-Self Report
RR: Risk ratio
LCA: Latent class analysis
OR: Odds ratio
BIC: Bayesian information criterion

## References

[1] International Labour Office (ILO), Global Employment Trends for Youth 2017: Paths to a Better working future. International Labour Office, Geneva: 2017.

[2] Eurofound. Long-term unemployed youth: Characteristics and policy responses. Publications Office of the European Union, Luxembourg: 2017.

[3] Vancea M, Utzet M (2017). How unemployment and precarious employment affect the health of young people: a scoping study on social determinants. Scand J Public Health..

[4] Hammarström A, Janlert U (2000). Do early unemployment and health status among young men and women affect their chances of later employment?. Scand J Public Health.

[5] Helgesson M, Johansson B, Nordqvist T, Lundberg I, Vingård E (2013). Unemployment at a young age and later sickness absence, disability pension and death in native swedes and immigrants. Eur J Pub Health.

[6] Herbig B, Dragano N, Angerer P (2013). Health in the long-term unemployed. Dtsch Arztebl Int.

[7] Kelly E, McGuinness S (2015). Impact of the great recession on unemployed and NEET individuals’ labour market transitions in Ireland. Econ Syst.

[8] Gerard MD, Valsamis D, van der Beken W (2012). Why invest in employment?.

[9] Hale DR, Bevilacqua L, Viner RM (2015). Adolescent health and adult education and employment: a systematic review. Pediatrics..

[10] Janlert U (1997). Unemployment as a disease and diseases of the unemployed. Scand J Work Environ Health.

[11] Roelfs DJ, Shor E, Davidson KW, Schwartz JE (2011). Losing life and livelihood: a systematic review and meta-analysis of unemployment and all-cause mortality. Soc Sci Med.

[12] Montgomery SM, Cook DG, Bartley MJ, Wadsworth MEJ (1998). Unemployment, cigarette smoking, alcohol consumption and body weight in young British men. Eur J Pub Health.

[13] Böckerman P, Hyytinen A, Kaprio J, Maczulskij T (2018). If you drink, don’t smoke: joint associations between risky health behaviors and labor market outcomes. Soc Sci Med.

[14] Brook JS, Zhang C, Burke L, Brook DW (2014). Trajectories of cigarette smoking from adolescence to adulthood as predictors of unemployment status in the early 40s. Nicotine Tob Res.

[15] Caspi A, Wright BRE, Moffitt TE, Silva PA (1998). Early failure in the labor market: childhood and adolescent predictors of unemployment in the transition to adulthood. Am Sociol Rev.

[16] Clark C, Smuk M, Lain D, Stansfeld SA, Carr E, Head J, Vickerstaff S (2017). Impact of childhood and adulthood psychological health on labour force participation and exit in later life. Psychol Med.

[17] Landhuis CE, Perry DK, Hancox RJ (2012). Association between childhood and adolescent television viewing and unemployment in adulthood. Prev Med.

[18] Rodwell L, Romaniuk H, Nilsen W, Carlin JB, Lee KJ, Patton GC (2018). Adolescent mental health and behavioural predictors of being NEET: a prospective study of adults not in employment, education, or training. Psychol Med.

[19] Heikkala E, Remes J, Paananen M, Taimela S, Auvinen J, Karppinen J (2014). Accumulation of lifestyle and psychosocial problems and persistence of adverse lifestyle over two-year follow-up among Finnish adolescents. BMC Public Health.

[20] McLeod JD, Uemura R, Rohrman S (2012). Adolescent mental health, behavior problems, and academic Archievement. J Health Soc Behav.

[21] Henriksson P, Henriksson H, Tynelius P, Berglind D, Löf M, Lee I-M, Shiroma EJ, Ortega FB (2019). Fitness and body mass index during adolescence and disability later in life: a cohort study. Ann Intern Med.

[22] Kaila-Kangas L, Haukka E, Miranda H, Kivekäs T, Ahola K, Luukkonen R, Shiri R, Kääriä S, Heliövaara M, Leino-Arjas P (2014). Common mental and musculoskeletal disorders as predictors of disability retirement among Finns. J Affect Disord.

[23] Miranda H, Kaila-Kangas L, Heliövaara M, Leino-Arjas P, Haukka E, Liira J, Viikari-Juntura E (2010). Musculoskeletal pain at multiple sites and its effects on work ability in a general working population. Occup Environ Med.

[24] Haukka E, Kaila-Kangas L, Ojajärvi A, Saastamoinen P, Holtermann A, Jorgensen MB, Karppinen J, Heliövaara M, Leino-Arjas P (2015). Multisite musculoskeletal pain predicts medically certified disability retirement among Finns. Eur J Pain.

[25] Heikkala E, Paananen M, Taimela S, Auvinen J, Karppinen J (2019). Associations of co-occurring psychosocial and lifestyle factors with multisite musculoskeletal pain during late adolescence – a birth cohort study. Eur J Pain.

[26] Mikkonen P, Leino-Arjas P, Remes J, Zitting P, Taimela S, Karppinen J (2008). Is smoking a risk factor for low back pain in adolescents? A prospective cohort study. Spine..

[27] Cole TJ, Bellizzi MC, Flegal KM, Dietz WH (2000). Establishing a standard definition for child overweight and obesity worldwide: international survey. BMJ..

[28] Achenbach TM, Rescorla LA (2001). Manual for the ASEBA school age forms and profiles.

[29] Laitinen J, Power C, Ek E, Järvelin MR (2002). Unemployment and obesity among young adults in a northern Finland 1966 birth cohort. Int J Obes Relat Metab Disord.

[30] Sutela E, Törmäkangas L, Toikka E, Haapakorva P, Hautakoski A, Hakovirta M, Rasinkangas J, Gissler M, Ristikari T. Nuorten hyvinvointi ja syrjäytymisen riskitekijät Suomen kuudessa suurimmassa kaupungissa: Helsinki, Espoo, Tampere, Vantaa, Oulu ja Turku. [The wellbeing of young adults, and risk factors contributing to social exclusion in the six largest cities in Finland: Helsinki, Espoo, Tampere, Vantaa, Oulu ja Turku.] Helsinki: Terveyden ja hyvinvoinnin laitos ja Turun yliopisto, 2016. https://www.julkari.fi/bitstream/handle/10024/130760/URN_ISBN_978-952-302-662-9.pdf.

[31] Kanerva N, Pietiläinen O, Lallukka T, Rahkonen O, Lahti J (2018). Unhealthy lifestyle and sleep problems as risk factors for increased direct employers’ cost of short-term sickness absence. Scand J Work Environ Health.

[32] Millman RP (2005). Working group on sleepiness in adolescents/young adults, APP committee on adolescence. Excessive sleepiness in adolescents and young adults; causes, consequences, and treatment strategies. Pediatrics..

[33] Sargent JD, Blanchflower DG (1994). Obesity and stature in adolescence and earnings in young adulthood. Arch Pediatr Adolesc Med.

[34] Ali SM, Lindström M (2006). Psychosocial work conditions, unemployment, and leisure-time physical activity: a population-based study. Scand J Public Health..

[35] Erskine HE, Norman RE, Ferrari AJ, Chan GCK, Copeland WE, Whiteford HA, Scott JG (2016). Long-term outcomes of attention-deficit/hyperactivity disorder and conduct disorder: a systematic review and meta-analysis. Am Acad Child Adolesc Psychiatry.

[36] Hakulinen C, Elovainio M, Pulkki-Råback L, Böckerman P, Viinikainen J, Pehkonen J, Raitakari OT, Keltikangas-Järvinen L, Hintsanen M (2016). Depressive symptoms and long-term income: the young Finns study. J Affect Disord.

[37] Yang X, Telama R, Hirvensalo M, Viikari JSA, Raitakari OT (2010). Sustained involvement in youth sports activities predicts reduced chronic job strain in early midlife. JOEM..

[38] Kari JT, Tammelin TH, Viinikainen J, Hutri-Kähönen N, Raitakari OT, Pehkonen J (2016). Childhood physical activity and adulthood earnings. Med Sci Sports Exerc.

[39] van den Berg TIJ, Elders LAM, de Zwart BCH, Burdorf A (2009). The effect of work-related and individual factors on the work ability index: a systematic review. Occup Environ Med.

[40] Mawn L, Oliver EJ, Akhter N, Bambra CL, Torgerson C, Bridle C, Stain HJ (2017). Are we failing young people not in employment, education or training (NEETs)?. A systematic review and meta-analysis of re-engagement interventions Systematic Reviews.

[41] Rongen A, Robroek SJ, van Lenthe FJ, Burdorf A (2013). Workplace health promotion: a meta-analysis of effectiveness. Am J Prev Med.

[42] Hollederer A (2018). Health promotion and prevention among the unemployed: a systematic review. Health Promot Int.

[43] Nevanperä N, Seitsamo J, Ala-Mursula L, Remes J, Hopsu L, Auvinen J, Tammelin T, Järvelin M-R, Laitinen J (2016). Perceived work ability in the light of long-term and stress-related unhealthy Behaviorsa – prospective cohort study. Int J Behav Med.

